# Using the social entrepreneurship approach to generate innovative and sustainable malaria diagnosis interventions in Tanzania: a case study

**DOI:** 10.1186/1475-2875-9-42

**Published:** 2010-02-03

**Authors:** Lisa K Allen, Erin Hetherington, Mange Manyama, Jennifer M Hatfield, Guido van Marle

**Affiliations:** 1 Department of Community Health Sciences, Faculty of Medicine, University of Calgary, 3330 Hospital Drive NW, Calgary AB, T2N 4N1, Canada; 2 Bugando University College of Health Sciences, P.O. Box 1464, Mwanza, Tanzania; 3 Department of Microbiology and Infectious Diseases, Faculty of Medicine, University of Calgary, 3330 Hospital Drive NW, Calgary AB, T2N 4N1, Canada

## Abstract

**Background:**

There have been a number of interventions to date aimed at improving malaria diagnostic accuracy in sub-Saharan Africa. Yet, limited success is often reported for a number of reasons, especially in rural settings. This paper seeks to provide a framework for applied research aimed to improve malaria diagnosis using a combination of the established methods, participatory action research and social entrepreneurship.

**Methods:**

This case study introduces the idea of using the social entrepreneurship approach (SEA) to create innovative and sustainable applied health research outcomes. The following key elements define the SEA: (1) identifying a locally relevant research topic and plan, (2) recognizing the importance of international multi-disciplinary teams and the incorporation of local knowledge, (3) engaging in a process of continuous innovation, adaptation and learning, (4) remaining motivated and determined to achieve sustainable long-term research outcomes and, (5) sharing and transferring ownership of the project with the international and local partner.

**Evaluation:**

The SEA approach has a strong emphasis on innovation lead by local stakeholders. In this case, innovation resulted in a unique holistic research program aimed at understanding patient, laboratory and physician influences on accurate diagnosis of malaria. An evaluation of milestones for each SEA element revealed that the success of one element is intricately related to the success of other elements.

**Conclusions:**

The SEA will provide an additional framework for researchers and local stakeholders that promotes innovation and adaptability. This approach will facilitate the development of new ideas, strategies and approaches to understand how health issues, such as malaria, affect vulnerable communities.

## Background

### Malaria

It is reported that 3.3 billion people live in areas where they are at risk of being infected with malaria [1]. Of these, each year more than 1 million people die due to illnesses that are diagnosed as malaria [2]. Since the clinical symptoms of malaria are unspecific, reliable laboratory malaria testing that is trusted by physicians is essential to ensure proper diagnosis and treatment [3]. Due to the lack of access to quality diagnostic facilities, clinicians in many African countries, including Tanzania, have to base their diagnosis purely on clinical features [4]. This commonly results in over-diagnosis and treatment, which is a major issue when considering the rising cost of effective combination anti-malarial drug therapy and the alarming increase of drug resistant malaria [5]. Consequently, reliable diagnostic testing paradigms are an essential element in the fight against malaria.

### Malaria diagnosis

In Tanzania, microscopy is the "gold standard" laboratory tool used to diagnose malaria [6]. The strength of microscopy as diagnostic tool is that it can quantify the disease through total parasite counts and can differentiate between parasite species [7]. This is important since treatment for malaria varies between parasite species and level of parasitaemia. However, microscopy requires properly trained technicians and properly maintained equipment and reagents, which are unavailable in many of the rural areas of Tanzania [8]. To date, there have been several vertical interventions in malaria endemic areas attempting to improve the accuracy of malaria diagnosis [9-11].

#### Interventions and the call for participatory research

Unfortunately, many vertical interventions have had limited long-term success, due to lack of local stakeholder/community engagement, limited integration of multiple disciplines and strategies, development of programmes 'out of context' and the resulting lack of evidence to influence policy [12]. Tanzanian health professionals acknowledge these short comings, and have called for applied participatory research that address problems with malaria diagnosis in a nature that is tailored specifically to the local clinical environment. This research approach is based upon collaboration with local stakeholders and understanding of local priorities.

This paper seeks to provide an additional concrete framework for research aimed to improve accuracy in malaria diagnosis using a hybrid of established methods from social sciences (participatory action research) and the business model aspects of social entrepreneurship. It is thought that this strategy will generate innovative and sustainable long-term outcomes that can be used to address the problem of accurate malaria diagnosis at the local clinical level.

### Participatory action research (PAR)

Participatory action research is a unique and important approach to public health as it is based on reflection, data collection and actions with the aim to reduce inequities and improve health through involvement and resulting empowerment of communities and individuals [13]. PAR is a methodology that promotes researchers to create partnerships with communities in order to promote positive social change. The advantages of PAR are that it is applied collaborative research created through use of a committed community. Furthermore, the topic of research originates from the community itself. The disadvantages to PAR are that it often has no defined research leader, may be impractical to achieve consensus from the community and usually has no defined timeline or set end date [14].

### Social entrepreneurship

Social entrepreneurship is based on an concept introduced by Bill Drayton over 25 years ago with the idea in mind that the collaboration of a global community are far more powerful than the sum of its solo practitioner parts [15-17]. In comparison to classical entrepreneurs, social entrepreneurs have as their central goal, societal impact, with capital wealth creation a secondary consideration [18,19]. Success for social entrepreneurs is measured in the ability to innovate, facilitate and sustain positive changes and growth for a defined social problem [18,20,21].

The concept of social entrepreneurship provides an additional framework for those engaging in applied health research and provides a unique focus on innovation and adaptation, which is not necessarily stressed in PAR. Furthermore, social entrepreneurship follows a structured timeline, which includes amongst others, specific milestones indicative of project success. The presence of a timeline may be helpful when attempting to deal with one of the disadvantages of PAR.

### Social entrepreneurship approach (SEA) to health research

It is anticipated that the social entrepreneurship approach (SEA), which is a combination of PAR and social entrepreneurship, can be used as a mechanism within health research in order to create sustainable change by incorporating important aspects such as a multi-disciplinary approach, constant innovation and adaptation, a defined timeline, as well as partnerships with key local stakeholders. The SEA is designed for the development and implementation of applied research projects in low to middle income countries (LMICs). The outcomes from this research may then be used as the building blocks for future community and clinical level interventions.

The current report does not intend to compare PAR and SEA in order to determine if one approach is better than the other. The purpose is to highlight the aspects of SEA that may offer an advantage compared to PAR approach, based on experiences over the last four years at a rural hospital in northern Tanzania. It is proposed that SEA may assist in the generation of a sustainable change in malaria diagnosis. This unique approach will address gaps in the design of previous malaria interventions and can be used to generate successful and sustainable interventions for malaria diagnosis, other diagnostic platforms for infectious diseases and a variety of other health issues.

## Methods

### The social entrepreneurship approach (SEA) to applied health research

Although there is no universally accepted theoretical framework for social entrepreneurship, Dees [18] broke down the stages and has proposed the following framework for the social entrepreneur: (1) adopting a mission to create and sustain social value, (2) recognizing and relentlessly pursuing new opportunities that serve that mission, (3) engaging in a process of continuous innovation, adaptation and learning, (4) acting boldly without being limited by resources currently in hand, and (5) exhibiting a heightened sense of accountability to the community involved and for the outcomes created.

The proposed SEA approach incorporates an adaptation of these stages of social entrepreneurship, based on the unique characteristics of applied health research. For example, Dees' first stage is defined as "adopting a mission to create and sustain social value". Similar to applied health research, research questions or hypotheses are identified based on locally relevant topics of study, instead of mission statements. This stage has been modified to, "identification of a locally relevant topic and research plan". A detailed outline of changes to the five key elements of Dees' framework are described in Table 1. As defined by this project, the resulting five key SEA elements are: (1) identifying a locally relevant topic and research plan, (2) recognizing the importance of international multi-disciplinary teams and incorporation of local knowledge, (3) engaging in a process of continuous innovation, adaptation, and learning, (4) remaining motivated and determined to achieve sustainable long-term research outcomes, and (5) sharing and transferring ownership of the project with the international and local partner.

**Table 1:** Adaptations made to the established framework for social entrepreneurship in order to suit applied participatory health research initiatives in low to middle income countries (LMICs)

Stage/Element	Dees SEA Description	SEA for Health Research Description	Reason for Adaptation
**1**	Adopting a mission to create and sustain local value	Identifying a locally relevant topic and research plan	Health research revolves around the formation of research questions or a hypothesis

**2**	Recognizing and relentlessly pursuing new opportunities that serve that mission	Recognizing the importance of international multi-disciplinary teams and incorporation of local knowledge	A diverse and well trained team will allow for pursuit of new opportunities and a better understanding of our research outcomes

**3**	Engaging in a process of continuous innovation, adaptation, and learning	Engaging in a process of continuous innovation, adaptation, and learning	No adaptation required

**4**	Acting boldly without being limited by resources currently in hand	Remaining motivated and determined to achieve sustainable long-term research outcomes	Identifying the idea that local stakeholder motivation is a key element for intervention long-term success

**5**	Exhibiting a heightened sense of accountability to the community involved and for the outcomes created	Sharing and transferring ownership of the project with the international and local partner	Accountability to the community lies in knowledge translation to key local stakeholders and partnering with stakeholders to maintain long-term research outcomes

It is proposed that these elements are essential to create a change that will produce expected long-term outcomes in difficult low to middle income (LMIC) settings and provide stability for future interventions in the unique rural environment. It is also understood that many elements of the SEA have overlap with key concepts in PAR. This provides a backbone for the SEA that is founded on proven health research methodology (PAR). What makes the SEA unique is that it incorporates the entrepreneurial aspects of strong focus on vision, innovation and adaptability, which are applied by local stakeholders at the community or local clinic level.

### Case description

#### Location, population and malaria

The location forming the focus of this case report, is a small rural hospital located in northern Tanzania that serves a population of approximately 77,580 patients of which most are Maasai. The Maasai are semi-nomadic pastoralists who travel great distances to graze their cattle and are exposed to a range of altitudes from 1,000 meters near Lake Eyasi, up to over 3,000 meters near the top of Mount LeMakarot. This creates a unique situation for malaria transmission with patients accessing the hospital exposed to a range of medium to low transmission zones for malaria. Patients accessing the hospital for malaria diagnosis and treatment are given artemether-lumefantrine (AL) (when available) as outpatients and more severe cases are treated as in-patients with intravenous quinine. When AL is in low supply, the clinicians are forced to dispense sulphadoxine/pyrimethamine (SP), for which drug resistance is reported in Tanzania [22-24].

According to hospital records, in 2008 malaria was responsible for 38% and 49% of in-patient admission in patients younger than five and older than five years respectively. Malaria was identified as the causal agent for 45% of total deaths reported by the in-patient department. Malaria is also reported as the number one cause of outpatient department attendance (46%) in 2008. Most recently, in January of 2009, 61% of malaria investigations using microscopy were diagnosed as positive by the laboratory staff. These statistics are consistent with nationally reported values of outpatient attendance due to malaria (40%), as well as the reported prevalence rates (42.8%) in high transmission zones (< 600 m) in northern Tanzania [25,26].

However, these numbers are cause for investigation due to the location of the patient population within a low to medium transmission zone for malaria. Previous research in northern Tanzania reported parasite prevalence of 9.3% in low (>1,200 m) and 16.0% in medium (600 m to 1,200 m) transmission zones [25]. Consequently, the reported prevalence of malaria at the hospital is unexpectedly high. This factor alone points to the possibility that malaria over-diagnosis may be a significant issue in this setting. Therefore, it was felt to be a good site to study improvements in diagnostic practices using the SEA framework.

### The social entrepreneurship approach to health research: examining the key elements

#### Element #1

##### Topic identification and development of a research plan

**Figure 1:**
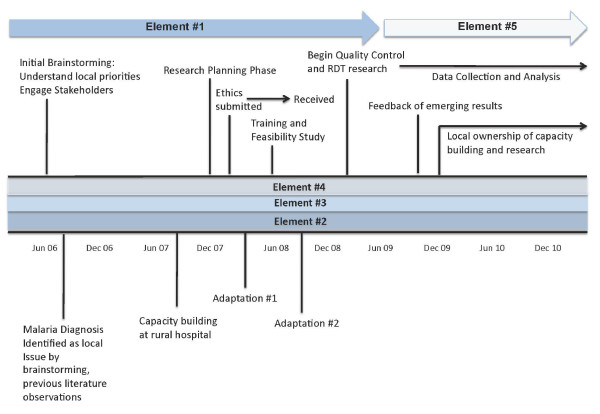
**Timeline of the Social Entrepreneurship Approach (SEA) key elements showing the corresponding major events and activities**. Elements 2-4 occur throughout the entire timeline of the applied research project. Element 1 is essential in the preparation and initiation of research. Element 5 occurs in the later phases of the applied research and is important for ensuring continued local ownership and motivation.

The health issue of local relevance was identified through a rigorous brainstorming session carried out during partnership-building activities at the Bugando University College of Health Sciences (BUCHS). Communication between collaborators with the essential local knowledge was maintained during all stages of development of the emerging research plan to ensure the local needs and context was addressed (Figure 1). The constant review of the literature helped identify previous research models used in similar locations that could act as initial frameworks [27,28].

The major emerging theme communicated by the partners in Tanzania and consistently recurring in the literature was the importance of accurate malaria diagnosis and treatment. The common consensus from the team was that the current "gold standard" of microscopy-based diagnosis of malaria generally does not produce consistently accurate results required to provide appropriate standard of care for patients. The World Health Organization (WHO) has recognized this and suggested a new strategy for diagnosing malaria in low resource areas, which incorporates using malaria rapid diagnostic tests (RDTs) as an alternate to microscopy when microscopy is not quality assured or services are unavailable. The problem with the proposed new strategy is that the accuracy of RDTs varies depending on location and malaria epidemiology [29]. Therefore, it is essential to evaluate specific RDTs within the setting they will be implemented and utilized in order to assure sensitivity and specificity.

Prior research and personal observations support the idea that in northern Tanzania, quality and availability of accurate malaria diagnosis using microscopy is below the nationally acceptable levels [30]. Consequently, a project with an aim to ensure the accuracy of microscopy and evaluate WHO-recommended alternative diagnostic methods, such as RDTs, was deemed appropriate for successful and sustainable malaria case management in the area.

##### Institutional and government ethical clearance

As with any research project, approval from research ethical boards in the collaborating Canadian Institution (Conjoint Health Research Ethics Board), The National Institute for Medical Research in Tanzania (NIMR) and the Tanzanian Commission for Science and Technology (COSTECH) were obtained. Engaging ethics committees from both developing and developed countries is essential in order to mitigate the risk of conducting research at a sub-optimal ethical standard [31]. However, apart from fundamental regulatory requirement it is also essential to the sustainability and uptake of research outcomes [32]. Through these approvals research outcomes will be linked directly to governmental organizations in Tanzania improving knowledge translation sustainability. The partnership with international and local institutions was critical in order to achieve the described formalities as well as when communicating with the respective ethics agencies to ensure approvals will be dealt with in a timely manner.

#### Element #2

Creating a team with a unified research objective and drawing on differing experience, knowledge and expertise to accomplish a set goal is critical for the success and sustainability of multi-disciplinary research. Two strategies were undertaken that included a broad range of biomedical, social science, international and local expertise. The strategies can be defined as: (1) engaging a multi-disciplinary approach to research, and (2) committing to an equitable partnership model.

##### Engaging a multi-disciplinary approach to research

A multi-disciplinary approach requires more than a collection of diverse experts working on the same topic, but rather a team of diverse experts who understand the importance of different perspectives and work together across disciplinary boundaries [33]. A multidisciplinary team, including molecular biologists, public health specialists, gender experts and physicians was utilized when planning and implanting this research. Planning and research development took place jointly and resulted in a multi-faceted approach to ensuring accurate malaria diagnosis. Not only was there a focus on laboratory techniques and new technologies, but also consideration of patient treatment-seeking behaviours, perceptions influencing laboratory analysis and physician behaviour. Cross-training members of the team through teaching of social science methods to molecular biologists and diagnostic techniques to physicians and social scientists was also considered critical for success, by facilitating conversations across disciplines.

##### Committing to an equitable partnership model

A focus on equitable partnerships is central to success of global health research [34]. Often research partnerships, especially international ones, are characterized by power differences between partners. This can lead to local partners being undervalued in the research process, acting only as host institutions and not contributing significantly to the research process. From the beginning, this project used local knowledge and expertise to develop the research topic. At each stage in the project, this approach relied on local expertise. For example, an internal and external quality control programme at the rural hospital in northern Tanzania was established prior to the initiation of research objectives (described in detail in the next section). Recognizing that Tanzanian scientists had high level of expertise in microscopy diagnosis of malaria, the partners at BUCHS became the external quality control laboratory for the rural hospital. This approach supports the idea that equitable partnerships will not only improve the quality of the research, but also help ensure its sustainability and create a sense of ownership to local stakeholders.

#### Element #3

##### Importance of innovation and adaptation

This element facilitates the most unique combination of social entrepreneurship and PAR. The entrepreneurial lens provides the vision and drive to continually innovate and adapt research approaches and objectives. PAR provides a grounded understanding of local priorities and influences that drive the constant innovation. Together, these approaches enable the creation of novel research outcomes that are accepted, adopted and hence sustainable in unique and ever changing environments.

In order to achieve element #3, considerable time was spent within Tanzania working with hospital staff and other key stakeholders to ensure the intended research: (1) addressed the identified issue of importance in a holistic manner, (2) did not add considerable burden to the hospital staff, (3) addressed an issue that was still current and in the forefront, (4) had clearly established priorities and action plan, and (5) was acceptable to all key stakeholders. Following the preliminary feasibility assessment, adaptations to the original research plan were made (Figure 1), which illustrate the importance of adaptability.

#### Adaptation #1: addition of another research component

From a brainstorming session, an idea emerged that, although improvements in quality of microscopy based diagnosis of malaria may be made, this does not mean that physicians will automatically have increased trust in microscopy. A review of the literature revealed that physician decision-making is influenced by a number of economic, social and political factors and that currently there is a lack of trust by physicians in the quality of laboratory results in developing countries such as Tanzania [35,36]. In response to the emergence and grounding of this new idea, another specific aim was added in attempts to address this particular social factor that influences malaria diagnosis and patient care at the hospital.

#### Adaptation #2: changes following the loss of critical laboratory staff

The rural hospital presented in this case is located approximately four hours drive from the closest urban center. As a result it is very difficult to attract highly trained and qualified staff [37]. Salaries at this hospital are not supplemented in order to retain individuals and consequently the rate of staff turnover becomes an issue both for the maintenance of quality control and the sustainability of this research. During the training and implementation phase, a key individual in the laboratory terminated employment at the hospital. This left the project with no institutionally trained laboratory technicians and a significant language barrier with the remaining staff.

In response to this, the format of the standard operating procedures (SOPs), the plan for implementing portions of the malaria blood smear staining SOPs and the logistics of implementing the quality control programmes had to be altered in the following ways: (1) SOPs were re-written in Kiswahili at the education level of the remaining technicians, (2) extra time was spent on training, (3) a significant amount of feedback from the remaining technicians and hospital staff was incorporated, (4) use of Giemsa was not implemented, (5) quality control programmes were adjusted to increase simplicity and reduce time burden, and (6) a two-day training module for the existing and incoming technicians was created outlining all proposed changes to methodologies.

#### Element #4

##### Key stakeholder motivation and retention

The development and implementation of multi-disciplinary international research often faces unique challenges. Success requires: (1) development of a strong foundation through equitable partnerships and collaborative development of priorities (2) open and honest communication about priorities and ability to engage in research activities, (3) shared accountability to generate research outcomes that provide a positive contribution to the local and academic community. When key stakeholders are involved from conception of the partnership, there is a resulting increase in motivation to produce desired research outcomes that can be used for future change. In addition, it is expected that the continued involvement also will give the local stakeholders the skills, tools and mindset to start up additional and independent projects targeting other issues relevant to their local needs.

##### Funding and cost

Funding opportunities were also identified through the traditional method of applying for grant funding from national or international agencies. Funding agencies targeted for institutions in Canada and Tanzania were identified and all proposals were generated collaboratively to strengthen the depth of academic and local knowledge. However, in the current economic environment it is increasingly important to search for funding opportunities that are outside convention, which fits very well with entrepreneurial philosophy underlying the SEA. Consequently, one must gather all potential resources, including people, money, volunteers and premises to "make a difference" [15]. For this project, funding searches began with identified venture capital funding opportunities. In response, a short project description with a purpose of pitching our project to the potential investors was generated, similar to strategies used in the business world. Materials were then distributed to potential investors along with a "business" pitch given by key leaders from the University of Calgary team to interested parties. The Tanzanian partners were also encouraged and taught to use similar approaches as possible means of generating funds to support their research and make their implemented changes sustainable.

The SEA approach has a number of hidden costs due to the extended timeline (Figure 1). The process of partnership building and research design based on locally identified priorities is a long-term investment, where research does not usually commence in the first year of the partnership. Considerable human resources and money is invested during this initial period often without the production of measurable outcomes. The benefit of the initial investment is exemplified in the later years of research, as outcomes are locally relevant and likely to have a positive and long-term influence on the development of interventions for malaria.

#### Element #5

##### Sustainability of research through training of local partners

A responsive rather than a prescriptive approach to team member training should be employed when engaging in collaborative research projects in developing countries such as Tanzania [37]. As a result, all training modules were developed following consultation with the director, clinical officers and laboratory staff at the local hospital. From these consultations it was determined that training material focusing on: (1) microscopy of malaria, (2) internal and external quality control programmes, and (3) the overall malaria research project design, would be prepared and delivered to the local hospital staff.

All training materials were developed for an interactive delivery. Materials were intended to engage the learner through question and answer sections. These can then be used as a seminar material as well as an individual learning tool. The 'train the trainer model' was used as a teaching style with the aim that local team members trained may then be able to train individuals entering the project over it's projected five year timeline and beyond. This method of training not only teaches the primary individual the skills and knowledge, but also trains the individual to transfer the knowledge to others, thus continuing the learning cycle.

##### Long-term sustainability of social change

This approach supports the idea that active participation in planning and implementation phases of applied research by the local partner will facilitate motivation and determination. The team agreed that the local partner holds the key to successful sustainability of research outcomes in this setting. Further, a number of identified critical factors must be taken into consideration within this setting in order to facilitate uptake and sustainability of research outcomes: (1) Health policy makers must be made aware of the critical importance of diagnostic accuracy in the laboratory. Laboratories in Africa are often lacking critical infrastructure such as running water and power and there is a serious shortage of reagents and equipment [4]. If policy makers increase funding and educational opportunities for laboratories and their staff, changes like those introduced in this project will gain long-term sustainability independent of international partner reliance.(2) If Tanzanian colleagues call on the health system to increase support for accuracy in the diagnostic laboratory, appropriate patient care and improved resource allocation will result. If the government requires that all laboratories utilize nationally standardized SOPs and internal and external quality control programmes, as outlined by this project, the momentum of social change in the area of malaria diagnosis that this project initiated will be sustainable.(3) Finally, if laboratory capacity improves, physician attitudes towards the reliability of laboratory testing may change. New trust in the improved accuracy of laboratory based malaria diagnosis may then have an impact at the hospital level [36].

## Discussion and evaluation

When discussing the achievement of research outcomes using the social entrepreneurship approach, the term milestones are used broadly. A retrospective evaluation of the achievement of defined milestones for each SEA element was conducted according to the corresponding strengths and identified challenges (Table 2). It is important to highlight that each element does not exist in isolation and the success, or achievement of milestones for one element is intricately tied to a number of other elements. Consequently, if the identified milestones for Element 1 are not achieved, it can be assumed that there will be difficulty achieving milestones for Element 2, and so on. For example, if the research fails to address a topic of local relevance (Element 1), it will be difficult to obtain support from local stakeholders (Element 2) and ensure that there is a high level of motivation to sustain research outcomes (Element 4). Understanding the logical relationships between these elements reveals the benefit of using the social entrepreneurial approach as a guideline when developing and implementing applied research projects in LMICs.

**Table 2:** The strengths and challenges for each Social Entrepreneurship Approach (SEA) Element and the corresponding milestones achieved

SEA Element	Strengths	Challenges	Milestones
**Element #1**	• Able to ensure research addresses a priority topic • Partnership building at initial stages of research	• Time to initiate project • Ensuring that all voices are given equal weight	• Ethical approvals achieved • Research collaboratively implemented

**Element #2**	• Approach the health issue from multiple perspectives • Mitigates inherent power differences between partners	• Organization of a large international team • Time commitment to create and sustain equitable partnerships	• Strong partnership with BUCHS and local hospital • Multi-disciplinary training delivered to local and international partners

**Element #3**	• Flexibility allows the project to remain relevant • Initial feasibility assessment enhances likelihood of success	• Adaptations increase time to complete research • Ensure that the adaptation does not introduce bias	• Increased communication between physicians and laboratory • Recruitment of experienced laboratory technician

**Element #4**	• Emphasis on societal impact allows for alternate funding sources • Local ownership leads to stakeholder motivation and retention	• Funding streams are discipline and disease specific • Under-resourced and over-committed local institutions	• In-kind donations of materials and local and international staff time • Capacity built to support local ownership of research

**Element #5**	• High level of knowledge translation • Promotion of long term sustainability	• Difficulty mobilizing resources at the national level • Local constraints of time and money	• Trained all interested local staff in multi-disciplinary topics • Strengthening of south-south partnerships

The social entrepreneurship approach to applied health research draws upon PAR with a focus on innovation and adaptability from social entrepreneurship. This creates a unique fusion of methodologies based on local priorities and driven by local stakeholders, who have the vision to constantly adapt research objectives and approaches based on current needs. As proposed by the SEA, adaptation is a critical element to successfully tailor research outcomes in difficult LMIC settings. It is thought that the SEA will provide a framework for researchers and local stakeholders to "think outside of the box". This approach will facilitate the development of new ideas, new strategies and approaches to understand how health issues, such as malaria, affect vulnerable communities in different ways.

## Conclusions

Generation of a sustainable change in the area of malaria diagnosis is a complex and long-term initiative that involves changes at the laboratory, physician and health system levels. Sustainability of research outcomes in LMICs, such as Tanzania, may be guided by the five key SEA elements presented in this report. This alternative approach is grounded in the use of multi-disciplinary collaborative partnerships that understand and value local priorities through engagement local stakeholders, so that an innovative strategy for improving the accuracy of malaria diagnosis can be created.

The value of incorporating social entrepreneurship in an applied research framework is the emphasis on innovation, and the ability to develop solutions tailored specifically to the local clinical environment. For this setting, innovation resulted in a holistic view of malaria diagnosis that includes the patient, laboratory and physician, and follows the basis for follow up research activities. Research engaged in understanding malaria diagnosis at these three levels simultaneously has limited representation in the literature. Yet the relationship between these individuals may be actually a key component in the fight against malaria.

## Abbreviations

The following abbreviations are used: ACT: (Artemisinin Combination Therapy); LMIC: (Low to Middle Income Country); SEA: (Social Entrepreneurship Approach); SOP: (Standard Operating Procedure); WHO: (World Health Organization).

## Competing interests

The authors declare that they have no competing interests.

## Authors' contributions

LKA, JMH and MM are involved in the initiation and continuation of the partnership between the Bugando University College of Health Sciences in Tanzania and the University of Calgary in Canada and conducted on-the ground research activities in Tanzania. LKA and GVM made substantial contributions to the conception and design. LKA prepared initial drafts and GVM, JMH, EH and MM assisted with additional reflections for the SEA elements and revisions to the manuscript. All authors read and approved the final manuscript.
